# Downregulation of MYO1C mediated by cepharanthine inhibits autophagosome-lysosome fusion through blockade of the F-actin network

**DOI:** 10.1186/s13046-019-1449-8

**Published:** 2019-11-07

**Authors:** Yanhao Zhang, Xiuxing Jiang, Qin Deng, Ziyi Gao, Xiangyu Tang, Ruoqiu Fu, Jinjiao Hu, Yunong Li, Lirong Li, Ning Gao

**Affiliations:** 1 College of Pharmacy, Army Medical University, 30 Gaotanyan Street, Shapingba District, Chongqing, 400038 China; 2 Greater Philadelphia Pharmacy, Philadelphia, USA; 3 Biomedical Analysis Center, Army Medical University, Chongqing, 400038 China; 4 Key Laboratory of Basic Pharmacology of Ministry of Education and Joint International Research Laboratory of Ethnomedicine of Ministry of Education, Zunyi Medical University, Zunyi, China

**Keywords:** Autophagy/Mitophagy, Cepharanthine (CEP), MYO1C, F-actin, Autophagosome-lysosome fusion

## Abstract

**Background:**

MYO1C, an actin-based motor protein, is involved in the late stages of autophagosome maturation and fusion with the lysosome. The molecular mechanism by which MYO1C regulates autophagosome-lysosome fusion remains largely unclear.

**Methods:**

Western blotting was used to determine the expression of autophagy-related proteins. Transmission electron microscopy (TEM) was used to observe the ultrastructural changes. An immunoprecipitation assay was utilized to detect protein-protein interactions. Immunofluorescence analysis was used to detect autophagosome-lysosome fusion and colocalization of autophagy-related molecules. An overexpression plasmid or siRNA against MYO1C were sequentially introduced into human breast cancer MDA-MB-231 cells.

**Results:**

We show here that cepharanthine (CEP), a novel autophagy inhibitor, inhibited autophagy/mitophagy through blockage of autophagosome-lysosome fusion in human breast cancer cells. Mechanistically, we found for the first time that MYO1C was downregulated by CEP treatment. Furthermore, the interaction/colocalization of MYO1C and F-actin with either LC3 or LAMP1 was inhibited by CEP treatment. Knockdown of MYO1C further decreased the interaction/colocalization of MYO1C and F-actin with either LC3 or LAMP1 inhibited by CEP treatment, leading to blockade of autophagosome-lysosome fusion. In contrast, overexpression of MYO1C significantly restored the interaction/colocalization of MYO1C and F-actin with either LC3 or LAMP1 inhibited by CEP treatment.

**Conclusion:**

These findings highlight a key role of MYO1C in the regulation of autophagosome-lysosome fusion through F-actin remodeling. Our findings also suggest that CEP could potentially be further developed as a novel autophagy/mitophagy inhibitor, and a combination of CEP with classic chemotherapeutic drugs could become a promising treatment for breast cancer.

## Background

Autophagy is an evolutionarily conserved, intracellular self-defense mechanism in which organelles and proteins are sequestered into autophagic vesicles and are subsequently degraded through fusion with lysosomes [1]. Mitophagy represents the selective engulfment of damaged mitochondria by autophagosomes, forming mitophagosomes, and their subsequent catabolism by lysosomes [2]. Autophagosome-lysosome fusion, a highly regulated process at the protein, lipid, and biochemical levels, depends on a variety of different factors, including RAB7, SNARE, lipids, BNIP3, INPP5E, HDAC6, calcium ions, and the cytoskeleton [3–8]. MYO1C is a slow monomeric actin-based motor protein adapted for sustained power mobility that links membrane cargo enriched in phospholipids, such as phosphatidylinositol 4,5-bisphosphate, to the actin cytoskeleton [9, 10]. Previous studies have shown that MYO1C plays a critical role in regulating membrane fusion during endocytosis and exocytosis [9, 11–14]. It has recently been reported that MYO1C plays a critical functional role in regulating autophagosome-lysosome fusion. Depletion of MYO1C causes an accumulation of autophagic structures caused by a block in lysosome fusion [15]. Increasing evidence shows that actin cytoskeletal proteins are essential for membrane fusion. Lysosomes require actin filaments on their surface for fusion with autophagosomes [7]. Since MYO1C is known to drive localized remodeling of the actin cytoskeleton at the cell surface [11, 16–18], it is likely that MYO1C may mediate autophagosome-lysosome fusion through remodeling of the actin cytoskeleton. However, the molecular mechanism by which MYO1C mediates autophagosome-lysosome fusion by actin remodeling remains poorly understood.

**Fig. 1:**
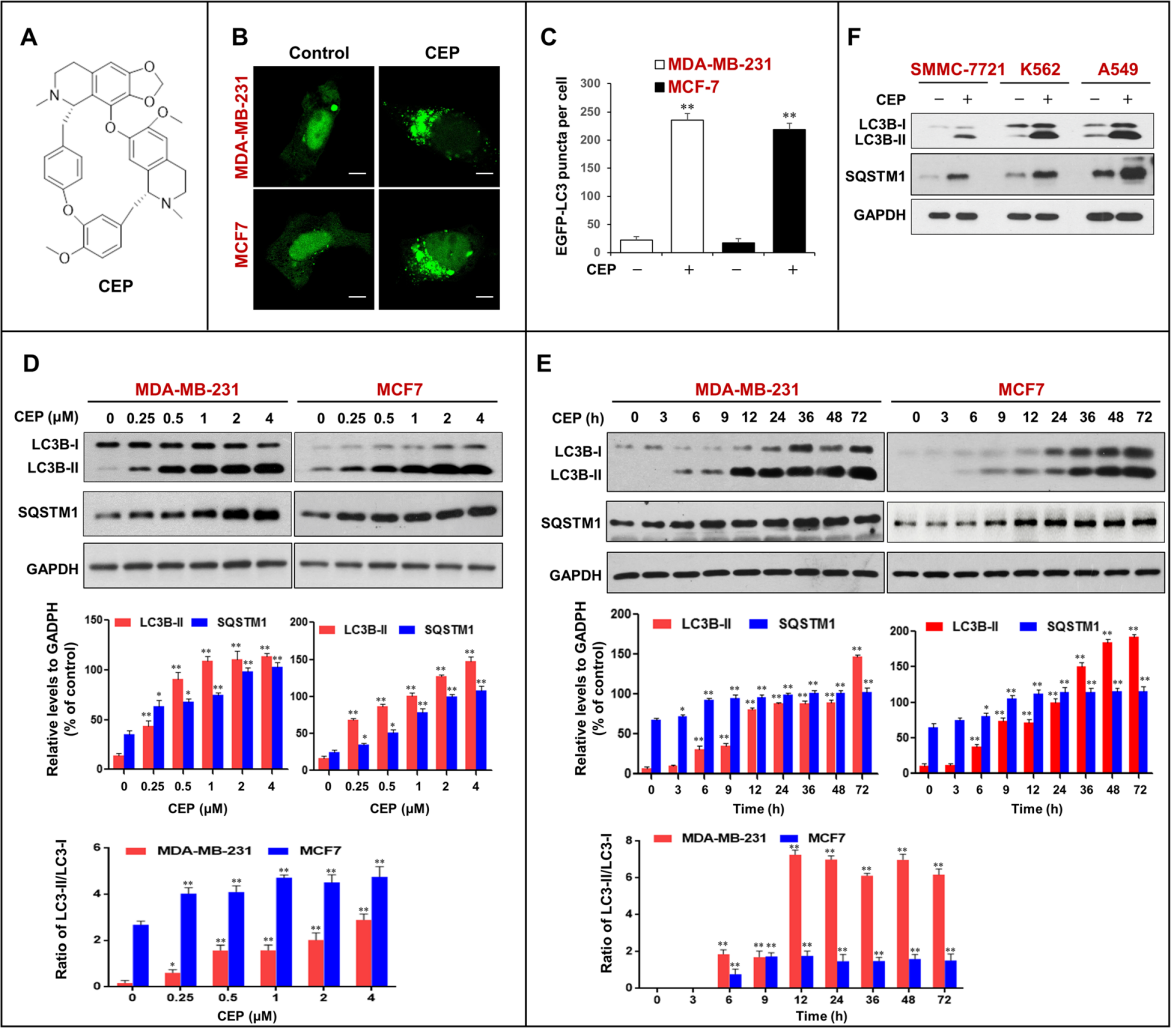
CEP triggers the accumulation of autophagosomes in human cancer cells. **a** The chemical structure of CEP. **b** MDA-MB-231 and MCF-7 cells transfected with EGFP-LC3 were treated without or with CEP (4 μM) for 24 h, the EGFP-LC3 puncta were observed under confocal microscopy; scale bars: 10 μm. **c** Quantification of average EGFP puncta per cell in (**b**) from 3 independent experiments. Data was presented as mean ± SD (**P < 0.01 compared with control); 50 cells were analyzed per treatment condition. **d** and **e** Cells were treated with various concentrations of CEP for 24 h, or treated with CEP (4 μM) for different time intervals as indicated. Western blot analysis was employed to determine the expression of autophagy-related proteins (LC3B-I/LC3B-II and SQSTM1). The relative levels of LC3B-II and SQSTM1 were normalized to GAPDH in three independent experiments (mean ± SD, *P < 0.05 or **P < 0.01 compared with control). The ratio of LC3-II/LC3-I was quantified in three independent experiments (mean ± SD, *P < 0.05 or **P < 0.01 compared with control). **f** Various cancer cells were treated with CEP (4 μM) for 24 h as indicated. The expression of LC3B-I/LC3B-II and SQSTM1 was detected by western blot. GAPDH was used as a loading control

Cepharanthine (CEP) is a benzylisoquinoline alkaloid extracted from *Stephania cepharantha* Hayata (Fig. 1a) [19]. CEP has been reported to exert a wide range of pharmacological effects, such as anti-inflammatory, antiviral, antimalarial, and anticancer effects. CEP has been shown to display diverse anticancer activities, including inhibition of cell proliferation, induction of apoptosis, anti-angiogenesis, anti-metastasis, etc. [20–23]. Recently, CEP has been found to exhibit anticancer effects through the modulation of autophagy [24]. Several studies have revealed that CEP induces autophagy and apoptosis in various types of cancer cells through the AMPK/mTOR or Akt/mTOR signaling pathways [25]. Tang ZH, et al. identified CEP as an autophagy inhibitor that acted through blockage of autophagosome-lysosome fusion in non-small cell lung cancer cells [24]. However, the precise molecular mechanism by which CEP inhibits autophagy through blockage of autophagosome-lysosome fusion remains largely unclear.

In the present study, we found that CEP inhibited autophagy/mitophagy by blocking autophagosome-lysosome fusion in human breast cancer cells. A mechanistic study revealed that MYO1C plays an important role in mediating autophagosome-lysosome fusion. Downregulation of MYO1C mediated by CEP inhibited autophagosome-lysosome fusion through inhibition of the F-actin network. The functional role of MYO1C in regulating autophagosome-lysosome fusion was further confirmed by genetic manipulation of MYO1C expression (i.e., knockdown or overexpression of MYO1C). Our findings suggest that CEP could potentially be further developed as a novel autophagy/mitophagy inhibitor, and a combination of CEP with classic chemotherapeutic drugs could represent a novel therapeutic strategy for the treatment of breast cancer.

## Materials and methods

### Cell culture

MDA-MB-231, MCF-7, K562, and A549 cells were provided by the American Type Culture Collection (ATCC, Manassas, VA). SMMC-7721 cells were obtained from the Bena Culture Collection (Beijing, China). Cells were cultured in DMEM (8118240, Gibco, USA), RPMI-1640 medium (8118021, Gibco, USA) or IMDM (AC10447366, HyClone, USA) containing 10% fetal bovine serum (10099133, Gibco, USA) at 37 °C in 5% CO_2_. 293FT cells (R70007, Invitrogen, USA) were cultured in DMEM containing 10% FBS, 0.5 mg/ml G418 (A1720-1G, Sigma-Aldrich, USA), 4 mM L-glutamine (1750007, Gibco, USA), 0.1 mM MEM nonessential amino acids (11140050, Gibco, USA), and 1 mM sodium pyruvate (11360070, Gibco, USA).

### Reagents and antibodies

Cepharanthine was purchased from Mansite Bio-Technology (A0653, Chengdu, China). Rapamycin (S1039, the treatment concentration was 0.25 μM) and bafilomycin A_1_ (S1413, the treatment concentration was 20 nM) were purchased from Selleck (Houston, TX, USA). Antibodies against SQSTM1 (5114, 1:1000 dilution), phospho-ULK1 (Ser757) (14,202, 1:1000 dilution), LAMP1 (9091, 1:1000 dilution), and LAMP2 (49,067, 1:1000 dilution) were obtained from Cell Signaling Technology (Boston, MA, USA); anti-GAPDH antibody (AG019, 1:1000 dilution) was obtained from Beyotime Biotechnology (Shanghai, China); and antibodies against actin (A1978, 1:5000 dilution), Beclin-1 (B6186, 1:5000 dilution), MYO1C (C82692, 1:1000 dilution) and LC3B (L7543, 1:5000 dilution) were from Sigma-Aldrich (Sigma, St. Louis, MO, USA).

### Transmission electron microscopy

After treatment as indicated, cells were fixed in 2.5% glutaraldehyde at 4 °C overnight, washed three times with PBS, and then postfixed with 1% osmium tetroxide for 2 h at room temperature. After fixation, the samples were dehydrated through a series of ethanol concentrations and embedded and stained with uranyl acetate/lead citrate. The sections were examined under a transmission electron microscope (JEM-1400PLUS, Japan).

### Preparation of the mitochondrial and cytosolic fractions

Mitochondrial and cytosolic fractions were isolated using a Cell Mitochondrial Isolation Kit (Beyotime Biotechnology, Shanghai, China) according to the manufacturer’s protocol. Briefly, cell pellets were washed with cold PBS and resuspended in 5× buffer A (20 mM HEPES, 10 mM KCl, 1.5 mM MgCl2, 1 mM EDTA, 1 mM EGTA, 1 mM Na3VO4, 2 mM leupeptin, 1 mM PMSF, 1 mM DTT, 2 mM pepstatin A, and 250 mM sucrose). For homogenization, the cells were passed through a 22-gauge needle 25 times. The homogenate was centrifuged at 4 °C in three sequential steps as follows: 1000 g, 10,000 g, and 100,000 g. The 10,000-g pellet was considered the “mitochondrial” fraction, and the 100,000 g supernatant was considered the “cytosolic” fraction. These fractions were subjected to western blot analysis.

### Western blotting and immunoprecipitation assay

To validate whether the band detection of western blots was within the linear range, the linear dynamic range of protein loading was determined. Cell lysates ranging from 5 μg to 80 μg protein were separated using SDS-PAGE and transferred to PVDF membranes (162–0177, Bio-Rad). Membranes were probed with antibodies as indicated above. The signal was detected using Clarity Western ECL Substrate (1,705,040, Bio-Rad). Quantitative detection of western blot bands was performed by densitometric analysis using ImageJ software. The correlation (R^2^) between densitometric intensity and protein load was calculated. A very good correlation (R^2^ > 0.95) to the two-fold dilutions was obtained in the typical range (between approximately 20 and 40 μg) of protein load, which was within the linear detection range of all antibodies. For all western blots, 20–40 μg of sample protein was used. The relative protein levels were normalized to GAPDH levels in three independent experiments. For immunoprecipitation, total protein lysates were obtained as described, and equal quantities of proteins were incubated with primary antibodies at 4 °C on a rocking platform. Immune complexes were collected with protein A/G agarose beads (88,802, Pierce) followed by 5 washes in PBS. Samples were then subjected to SDS-PAGE and western blot.

### Immunofluorescence

Cells were cultured on coverslips to 70% confluency and then transfected with plasmids for 48 h. After treatment, cells were prepared for immunostaining by incubation with primary antibodies and then incubated with secondary antibodies conjugated with Alexa Fluor 405 (A31553, 1:300), Alexa Fluor 488 (A11001, 1:300), or Alexa Fluor 647 (A31573, 1:300) (Molecular Probes, OR, USA) for 1 h at 37 °C. F-actin was stained with phalloidin (A12379, Invitrogen) at a 1:40 dilution. Mitochondria and lysosomes were stained with MitoTracker Red CMXRos (M7512, Molecular Probes) and LysoTracker Red DND 99 (L7528, Molecular Probes), respectively, according to the manufacturer’s instructions. Cells were viewed using a laser-scanning confocal microscope (Zeiss, Germany). All images were analyzed by ImageJ software (MD, USA).

### LC-MS analysis and protein identification

Cells were lysed in a buffer containing 8 M urea, 2 M thiourea, 2% 3-[(3-cholamidopropyl)dimethylammonio] propanesulfonate (CHAPS), 65 mM DTT, 1% nuclease mix, 1 mM NaF, 1 mM Na3VO4, 10 μg/mL aprotinin, 10 μg/mL leupeptin, and 1 mM PMSF. The whole cell lysate (100 μg) was digested with trypsin under standard conditions. Peptides were extracted and subjected to LC-MS analyses on LTQ-Orbitrap Velos Pro spectrometer (ThermoFisher Scientific) coupled to an Ultimate 3000 series liquid chromatography system using label free quantification (LFQ). The analysis of MS/MS data was performed using Proteome Discoverer (1.4.0) software against the UniProtKB *Homo sapiens* (Human) database.

### Transfections, RNA interference and MYO1C overexpression

Transfection was performed using Lipofectamine 3000 Transfection Reagent (L3000, Invitrogen) according to the manufacturer’s protocol. After transfecting cells with the plasmids for 24 h, the transfection mixture was removed and replaced with fresh complete medium. For RNA interference, cells were transfected with MYO1C siRNA from GeneChem Co Ltd. (Shanghai, China). The target sequences of MYO1C siRNAs were designed to target the indicated cDNA sequences: siRNA #1, 5′-AAG GCG TTG TAC AGC CGG ACA TT-3′ and siRNA #2, 5′-AAG CTT CCA GAC AGG GAT CCA TG-3′. A scrambled sequence (5′-CAG TCG CGT TTG CGA CTG G-3′) was used as a control**.** For MYO1C overexpression, cells were transfected with the MYO1C plasmid constructed by Gene Chem Co. Ltd. (Shanghai, China) according to the manufacturer’s protocol. After a 24 h incubation, the transfection mixture was removed and replaced with fresh complete medium for the experiment.

### Statistical analysis

Statistical analysis was performed with SPSS 20 software (SPSS, Chicago, Illinois, USA). Comparisons were performed using Student’s t-test or one-way analysis of variance (ANOVA). *P < 0.05, **P < 0.01 were considered statistically significant.

## Results

### CEP triggers the accumulation of autophagosomes/mitophagosomes in human cancer cells

To determine whether CEP could influence autophagy in human breast cancer cells, MDA-MB-231 and MCF7 cells were transiently transfected with EGFP-LC3, and the accumulation of autophagosomes was detected with a confocal laser-scanning microscope. As shown in Fig. 1b and c, treatment with CEP (4 μM) for 24 h resulted in an obvious increase in EGFP-LC3 puncta formation in these cells. Next, we examined the effects of CEP on the expression of LC3B-II (an autophagy marker) and SQSTM1 (an ubiquitin-binding receptor protein) using western blot analysis. Treatment with CEP caused dose- and time-dependent increases in the levels of LC3B-II or the ratio of LC3-II/LC3-I and SQSTM1 in MDA-MB-231 and MCF7 cells (Fig. 1d and e). Similarly, CEP treatment caused accumulation of LC3B-II and SQSTM1 in SMMC-7721 (a human hepatocellular carcinoma cell line), K562 (a human leukemia cell line), and A549 (a human lung cancer cell line) cells (Fig. 1f).

**Fig. 2:**
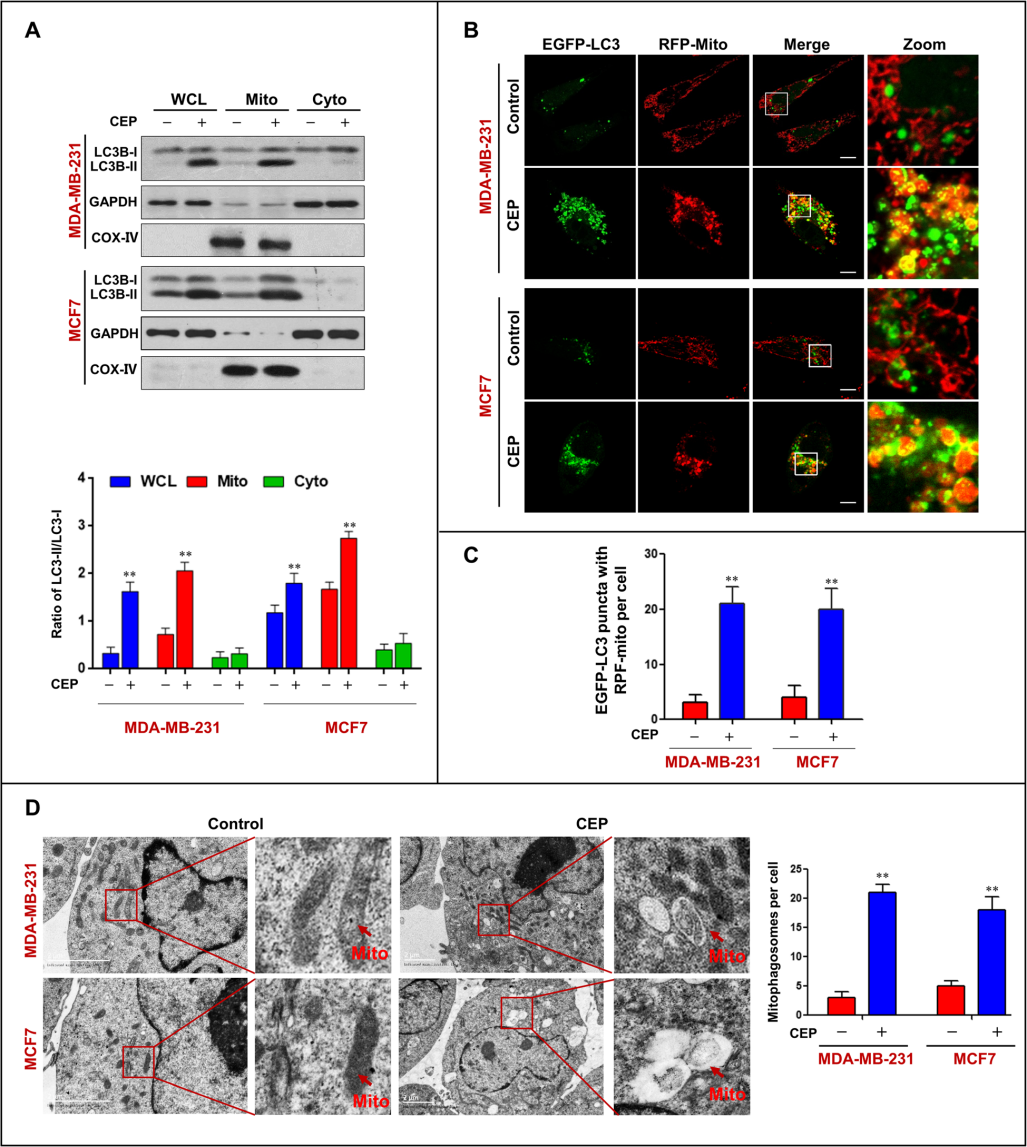
CEP triggers the accumulation of mitophagosomes in human breast cancer cells. MDA-MB-231 and MCF-7 cells were treated with CEP (4 μM) for 24 h. **a** Whole cellular lysates (WCL), mitochondrial (Mito) and cytosolic (Cyto) extracts were prepared and subjected to Western blot analysis using antibodies against LC3B-I/LC3B-II. GADPH and COX IV were used as a loading control. The ratio of LC3-II/LC3-I was quantified in three independent experiments (mean ± SD, *P < 0.05 or **P < 0.01). **b**-**c** Confocal microscopy images of cells treated without or with CEP (4 μM) for 24 h after co-expressing RFP-mito and EGFP-LC3; scale bars: 10 μm. Quantification of EGFP-LC3 and RFP-mito colocalization puncta in 50 cells of three independent experiments (**P < 0.01). **d** Representative TEM images of MDA-MB-231/MCF7 cells treated with or without CEP, red arrows indicate mitochondrial. Scale bars: 2 μm

To further determine the characteristics of autophagy in MDA-MB-231 and MCF-7 cells treated with CEP, the expression of LC3B-II in whole cell lysates and mitochondrial and cytosolic fractions was detected by western blot analysis. Interestingly, increased levels of LC3B-II were observed in the whole cell lysate and mitochondrial fraction of cells treated with CEP, but not in the cytosolic fraction (Fig. 2a). Immunofluorescence analysis showed that increased colocalization of EGFP-LC3 puncta and RFP-Mito was observed in cells treated with CEP (Fig. 2b and c). Using transmission electron microscopy, we clearly observed abnormal mitochondria that were fused with autophagic vesicles or surrounded by double membranes typical of autophagosomes (Fig. 2d). These findings suggest that CEP can influence the occurrence of mitophagy in human breast cancer cells.

### CEP inhibits autophagic flux in breast cancer cells

**Fig. 3:**
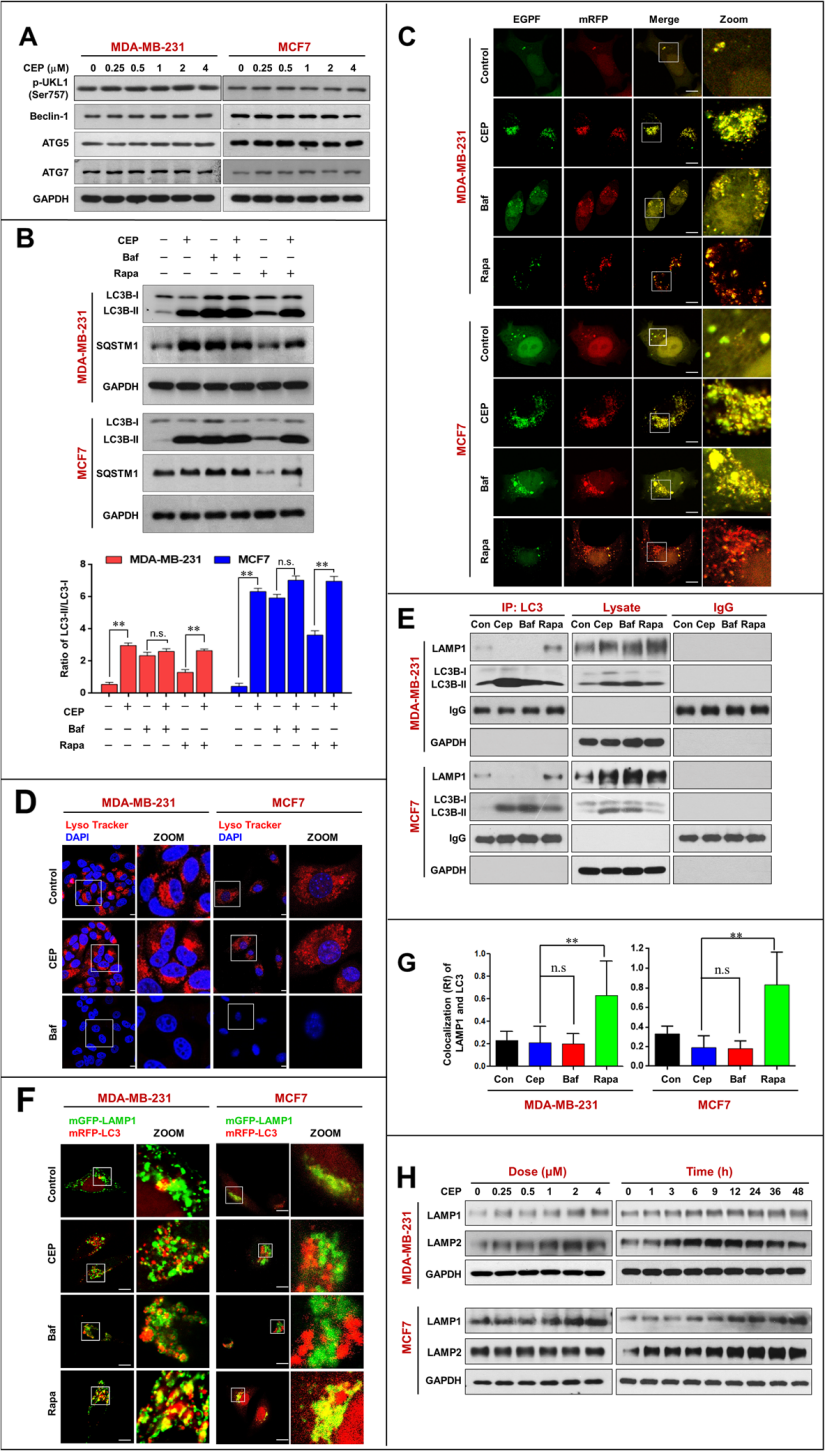
CEP inhibits autophagic degradation in MDA-MB-231 and MCF-7 cells by interfering autophagosome-lysosome fusion. **a** MDA-MB-231 and MCF-7 cells were exposed to various concentrations of CEP for 24 h. The expressions of autophagy-related proteins (p-ULK1(Ser757), Beclin-1, ATG5, and ATG7) were detected by western blot. **b** Cells were treated without or with CEP (4 μM) in the presence or absence of 20 nM bafilomycin A_1_ (Baf) or 0.25 μM rapamycin (Rapa) for 24 h, the expressions of SQSTM1 and LC3B-I/LC3B-II were analyzed by western blot. The ratio of LC3-II/LC3-I was quantified in three independent experiments (mean ± SD, *P < 0.05 or **P < 0.01). **c** Cells were transfected with a tandem reporter construct (tfLC3), and were exposed to CEP (4 μM), Baf (20 nM) and Rapa (0.25 μM). The colocalization of EGFP and mRFP-LC3 puncta was examined by confocal microscopy. Scale bars: 10 μm. **d** Cells were treated with CEP (4 μM) or Baf (20 nM) for 24 h. The fluorescent signals were detected by confocal microscopy after stained with LysoTracker Red. Scale bars: 10 μm. **e** Cells were treated with CEP (4 μM) or Rapa (0.25 μM) for 24 h, whole-cell lysate was prepared and subjected to immunoprecipitation using anti-LC3B and the associated LC3B-II and LAMP1 were determined using immunoblotting. **f** Cells cotransfected with mRFP-LC3 and LAMP1-mGFP were treated without or with CEP (4 μM), Baf (20 nM), or Rapa (0.25 μM). The colocalization of mRFP-LC3 and LAMP1-mGFP was detected by confocal fluorescent microscopy. Scale bars: 10 μm. **g** The Pearson’s correlation coefficient (R^2^) of LAMP1 and LC3 colocalization was from 50 cells of three independent experiments (mean ± SD from, n.s, not significant **P < 0.01). **h** Cells were exposed to various concentrations of CEP for 24 h, or treated with CEP (4 μM) for different time intervals as indicated. The expressions of LAMP1, LAMP2 were determined by western blot and GAPDH was used as a loading control

Autophagy is a cellular lysosomal degradation pathway that is essential for the regulation of cell survival and death to maintain cellular homeostasis [1]. A number of autophagy-related (ATG) proteins are key players in the process of autophagy [26]. We next investigated the effects of CEP on the levels of autophagy-related proteins. Treating cells with CEP did not affect the expression of p-ULK1 (Ser757), ATG5, ATG7, or Beclin-1 (Fig. 3a), suggesting that CEP does not cause autophagic vesicle nucleation and autophagosome formation.

Since our study showed that CEP treatment induced the expression of SQSTM1 in breast cancer cells, we speculated that CEP may act as a potent autophagic flux inhibitor and inhibit autophagic degradation. To test this possibility, we examined the effects of CEP on the accumulation of LC3B-II and SQSTM1 in the presence or absence of bafilomycin A_1_ (an autophagic flux inhibitor) or rapamycin (an autophagy inducer) by using western blot analysis. Combined treatment of CEP and bafilomycin A_1_ did not further increase the accumulation of LC3B-II and SQSTM1 caused by CEP. In contrast, treatment with rapamycin resulted in a modest increase in the levels of LC3B-II that were further enhanced by CEP. Furthermore, treatment with rapamycin led to decreased SQSTM1 levels, which was markedly reversed by CEP (Fig. 3b). These results indicate that CEP acts similarly to bafilomycin A_1_ by blocking autophagic degradation.

To further examine whether the effects of CEP are due to suppressed autophagic flux, MDA-MB-231 and MCF-7 cells transfected with a tandem reporter construct (tfLC3) were treated with CEP followed by assessment of EGFP-LC3 and mRFP-LC3 puncta colocalization. Similar to bafilomycin A_1_, treatment with CEP caused pronounced formation of LC3 puncta that displayed both green and red fluorescence producing a yellow overlay (Fig. 3c). In contrast, cells exposed to rapamycin led to the production of large amounts of red-only puncta (Fig. 3c). These findings suggest that CEP inhibits the late stage of autophagy, thereby resulting in a marked accumulation of autophagosomes.

### CEP inhibits autolysosome formation by interfering with autophagosome-lysosome fusion

Next, we determined the effect of CEP on lysosome function. The intralysosomal pH is a critical factor in determining lysosomal functions [27]. Thus, we examined the effect of CEP on intralysosomal pH by using LysoTracker Red for labeling and tracking acidic organelles [28]. Unfortunately, CEP treatment did not affect intralysosomal pH compared to the control, whereas bafilomycin A_1_ effectively abolished LysoTracker fluorescence (Fig. 3d), suggesting that CEP does not affect lysosomal acidification and may inhibit autolysosome formation through a different mechanism from that mediated by bafilomycin A_1_.

In the late stage of autophagy, fusion of autophagosomes with lysosomes leads to the formation of autolysosomes [29]. Inhibition of this process impairs autophagic degradation [30]. To address whether CEP affects autophagosome-lysosome fusion, we used immunoprecipitation analysis to determine the interaction of LC3 and LAMP1. As shown in Fig. 3e, treating MDA-MB-231 and MCF-7 cells with CEP decreased the interaction of LC3 and LAMP1. In contrast, treating cells with rapamycin increased the interaction of LC3 and LAMP1. Immunofluorescence analysis revealed that RFP-LC3 did not colocalize with LAMP1 in cells treated with CEP, which was similar to what was observed in cells treated with bafilomycin A_1_. In contrast, rapamycin treatment markedly increased the colocalization of LC3 and LAMP1 (Fig. 3f and g).

Since lysosome-associated proteins LAMP1 and LAMP2 are critical for autophagosome-lysosome fusion [31], we then examined the effects of CEP on the expression of these proteins using western blot analysis. Treating cells with CEP led to increased levels of LAMP1 and LAMP2 (Fig. 3h), suggesting that the blockade of autophagic flux mediated by CEP was not due to reduced expression of LAMP1 and LAMP2. Taken together, these findings suggest that CEP inhibits autophagy not by affecting lysosomal function but by impairing autophagosome-lysosome fusion.

### Downregulation of MYO1C is involved in the CEP-mediated inhibition of autophagy-lysosome fusion

**Fig. 4:**
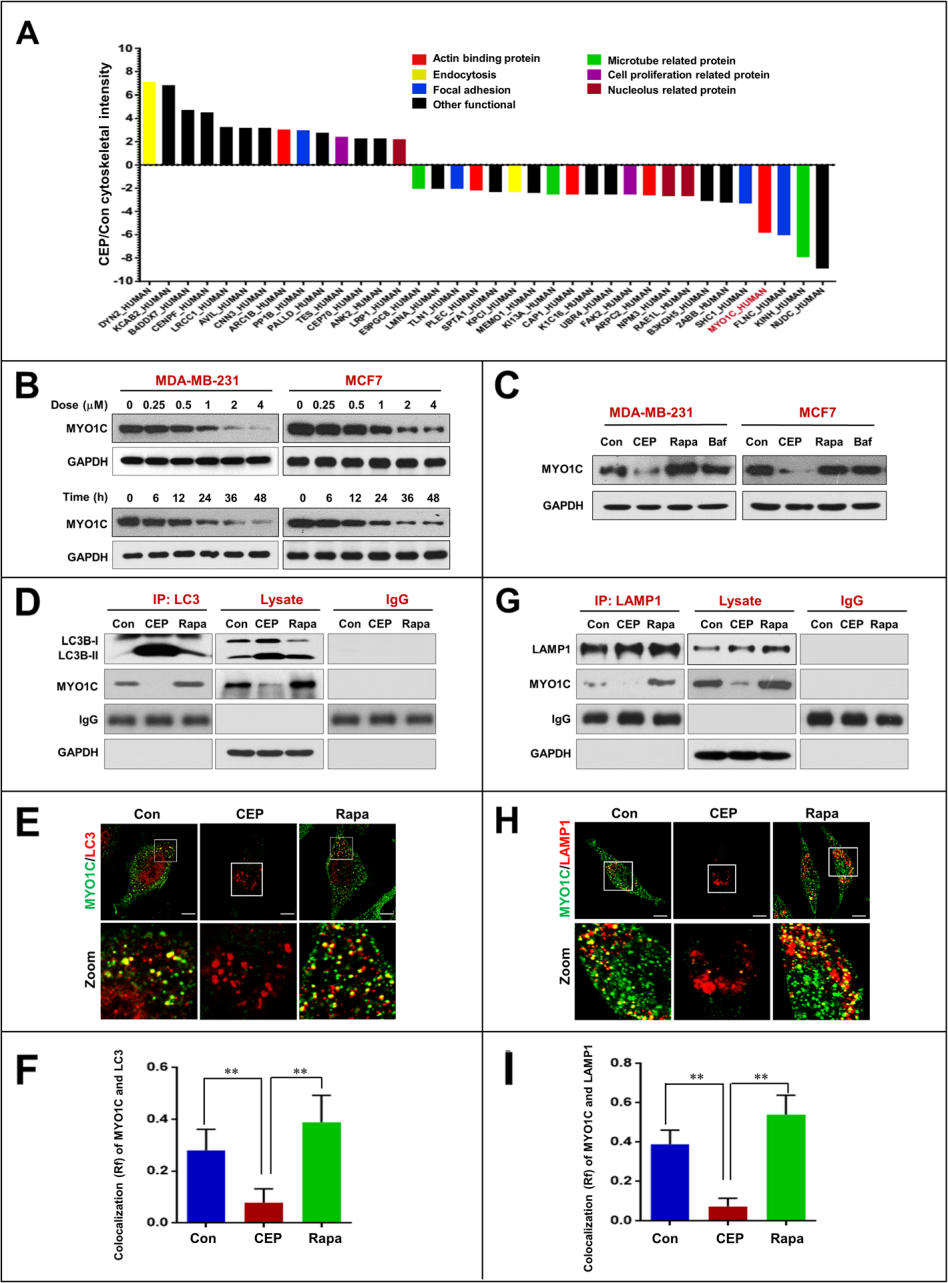
CEP downregulates MYO1C and inhibits the interaction/colocalization of MYO1C with LC3 and LAMP1. MDA-MB-231 cells were treated without or with CEP (4 μM) for 24 h, after which whole cell lysates were digested using trypsin and subjected to LC-MS to identify differentially expressed proteins. Each group consist three biological repeats. **a** 36 cytoskeleton related proteins upregulated or downregulated at least 2-fold were selected and then divided into seven group by DAVID Functional Annotation Bioinformatics Microarray Analysis. **b** Cells were treated with various concentrations of CEP for 24 h, or treated with CEP (4 μM) for different time intervals as indicated. The expression of MYO1C was determined by western blot analysis. **c** Cells were treated with CEP (4 μM) or Rapa (0.25 μM) or Baf (20 nM) for 24 h, after which the expression of MYO1C was detected by western blot analysis. **d** MDA-MB-231 cells were treated without or with CEP (4 μM) or Rapa (0.25 μM) for 24 h. Whole-cell lysate was prepared and subjected to immunoprecipitation using anti-LC3 and the associated LC3B-II and MYO1C were determined using immunoblotting. **e** The colocalization of LC3 and MYO1C was determined by confocal microscopy. **f** The Pearson’s correlation coefficient (R^2^) of MYO1C and LC3 colocalization 50 cells of three independent experiments (mean ± SD, *P < 0.05; **P < 0.01). **g** LAMP1 was immunoprecipitated, and the expression MYO1C was determined by western blot analysis. **h** The fluorescent images showed the colocalization of MYO1C with LAMP1. **i** The Pearson’s correlation coefficient (R^2^) of MYO1C and LAMP1 colocalization was from 50 cells of three independent experiments (mean ± SD, *P < 0.05; **P < 0.01)

Recent studies have revealed that several cytoskeletal components have essential roles in regulating autophagy [16]. Next, we employed a proteomic approach to identify which cytoskeletal component is involved in the inhibition of autophagosome-lysosome fusion mediated by CEP treatment. We treated MDA-MB-231 cells either with or without CEP and identified differentially expressed proteins by LC-MS analysis. We found that 36 cytoskeleton-related proteins were significantly altered in the histogram (Fig. 4a and Additional file 1: Table S1). It has recently been reported that loss of functional MYO1C disrupts autophagosome-lysosome fusion [15]. Our proteomic data also showed that MYO1C was significantly downregulated at least 5-fold in cells treated with CEP compared to control cells (Fig. 4a). Western blot analysis confirmed that treating cells with CEP resulted in noticeable decreases in the levels of MYO1C in dose- and time-dependent manners (Fig. 4b). Treatment with rapamycin resulted in a modest increase in the levels of MYO1C. However, treatment with bafilomycin A_1_ did not change the levels of MYO1C (Fig. 4c). These findings suggest that downregulation of MYO1C mediated by CEP may be involved in the blockade of autophagosome-lysosome fusion.

To further confirm that downregulation of MYO1C mediated by CEP treatment could be involved in the blockade of autophagosome-lysosome fusion, we next examined whether CEP affects the interaction of MYO1C with autophagosomes (LC3) by using immunoprecipitation analysis. As shown in Fig. 4d, the interaction of MYO1C and LC3 was decreased by CEP treatment but increased by rapamycin treatment. Similarly, immunofluorescence analysis revealed that the colocalization of MYO1C and LC3 was decreased by CEP treatment but increased by rapamycin treatment (Fig. 4e and f). We also used immunoprecipitation analysis to determine whether CEP affects the interaction of MYO1C with lysosomes (LAMP1). The interaction of MYO1C with LAMP1 was decreased by CEP treatment but increased by rapamycin treatment (Fig. 4g). Similarly, the colocalization of MYO1C with LAMP1 was decreased by CEP treatment but increased by rapamycin treatment (Fig. 4h and i). These findings suggest that the CEP-mediated blockade of autophagosome-lysosome fusion was due to impaired recruitment of MYO1C to both autophagosomes and lysosomes.

### CEP inhibits autophagosome-lysosome fusion through blockade of a MYO1C-dependent remodeling of the F-actin network

**Fig. 5:**
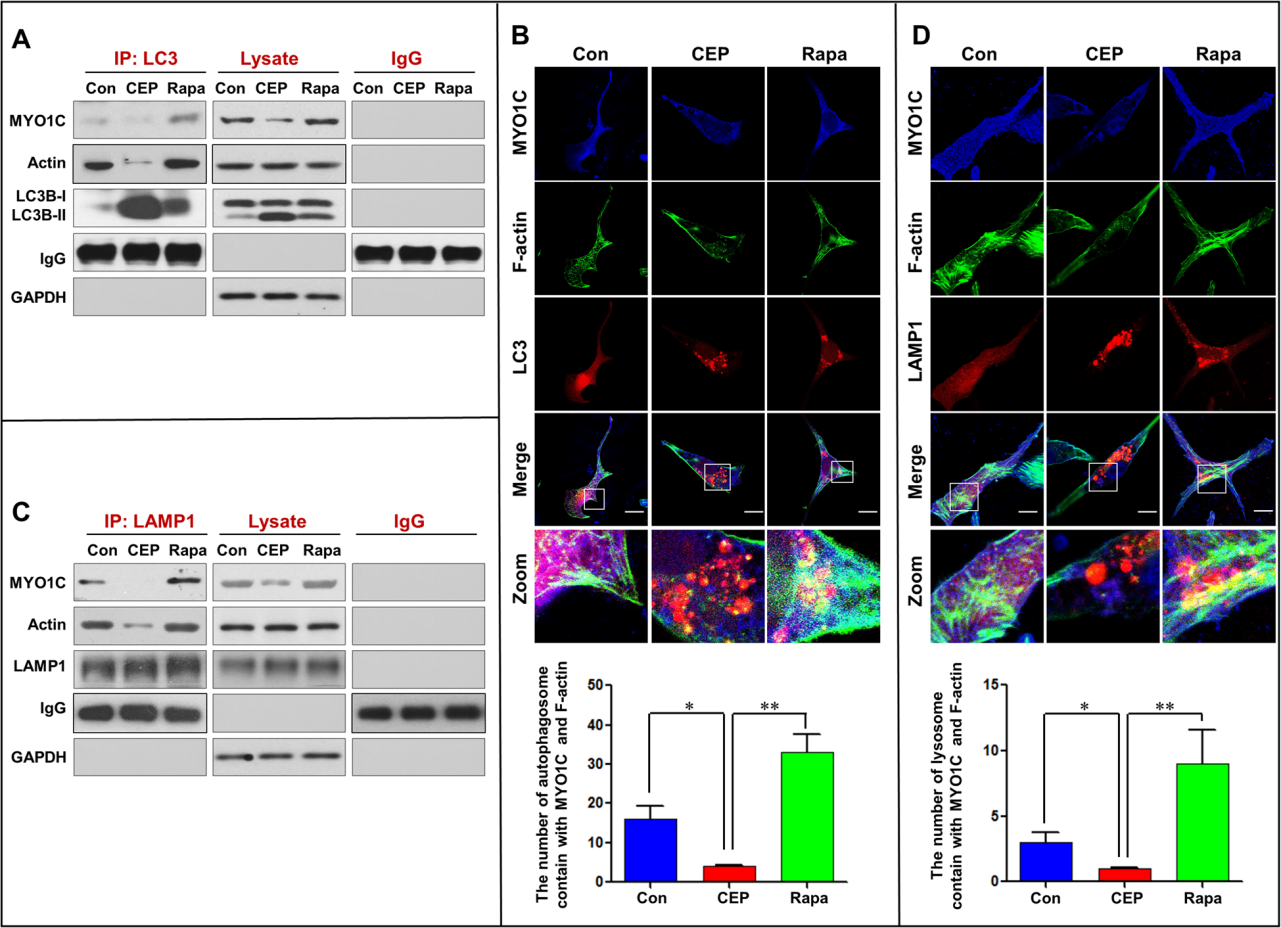
CEP disrupts the interaction/colocalization of MYO1C/F-actin with LC3 and LAMP1. MDA-MB-231 cells were treated without or with CEP (4 μM) or Rapa (0.25 μM) for 24 h. **a** Whole-cell lysate was prepared and subjected to immunoprecipitation using anti-LC3 and the associated MYO1C and actin were determined using immunoblotting. **b** The fluorescent images of MDA-MB-231 cells immunostained for MYO1C (blue), F-actin (green) and LC3 (red). **c** LAMP1 was immunoprecipitated, and the expressions of MYO1C and actin were determined by western blot analysis. **d** The fluorescent images of cells immunostained for MYO1C (blue), F-actin (green) and LAMP1 (red). **e** The number of LC3 puncta colocalized with MYO1C and F-actin was quantified from 50 cells in three dependent experiments, data was present as mean ± SD (*P < 0.05; **P < 0.01). **f** The number of LAMP1 puncta colocalized with MYO1C and F-actin was quantified from 50 cells in three dependent experiments, data was present as mean ± SD (*P < 0.05; **P < 0.01)

Recent evidence has revealed that the actin cytoskeleton is required for membrane fusion [32, 33]. Since MYO1C is an actin filament transporter that mediates actin filament movement to the cellular membrane [11, 34], we speculated that disruption of the MYO1C-dependent remodeling of the F-actin network may be involved in the CEP-mediated inhibition of autophagosome-lysosome fusion. To test this possibility, we examined whether CEP treatment affects the interaction of LC3 with MYO1C and actin by using immunoprecipitation analysis. CEP treatment obviously reduced the coprecipitation of LC3 with MYO1C and actin, whereas treatment with rapamycin increased the interaction of LC3 with MYO1C and actin compared to control treatment (Fig. 5a). Similarly, immunofluorescence analysis revealed that the colocalization of LC3 with MYO1C and F-actin was decreased by CEP treatment but increased by rapamycin treatment (Fig. 5b). Based on the above results showing that CEP treatment also decreased the interaction and colocalization of MYO1C with lysosomes (LAMP1), we next examined whether CEP affects the interaction and colocalization of LAMP1 with MYO1C and F-actin by using immunoprecipitation and immunofluorescence analyses. The interaction and colocalization of LAMP1 with MYO1C and F-actin were decreased by CEP treatment but increased by rapamycin treatment compared to those in control cells (Fig. 5c and d). These findings suggest that CEP inhibits autophagosome-lysosome fusion through blockade of a MYO1C-dependent assembly of the F-actin network to both autophagosomes and lysosomes.

### Depletion of MYO1C decreases autophagosome-lysosome fusion and increases the accumulation of mitophagosomes

**Fig. 6:**
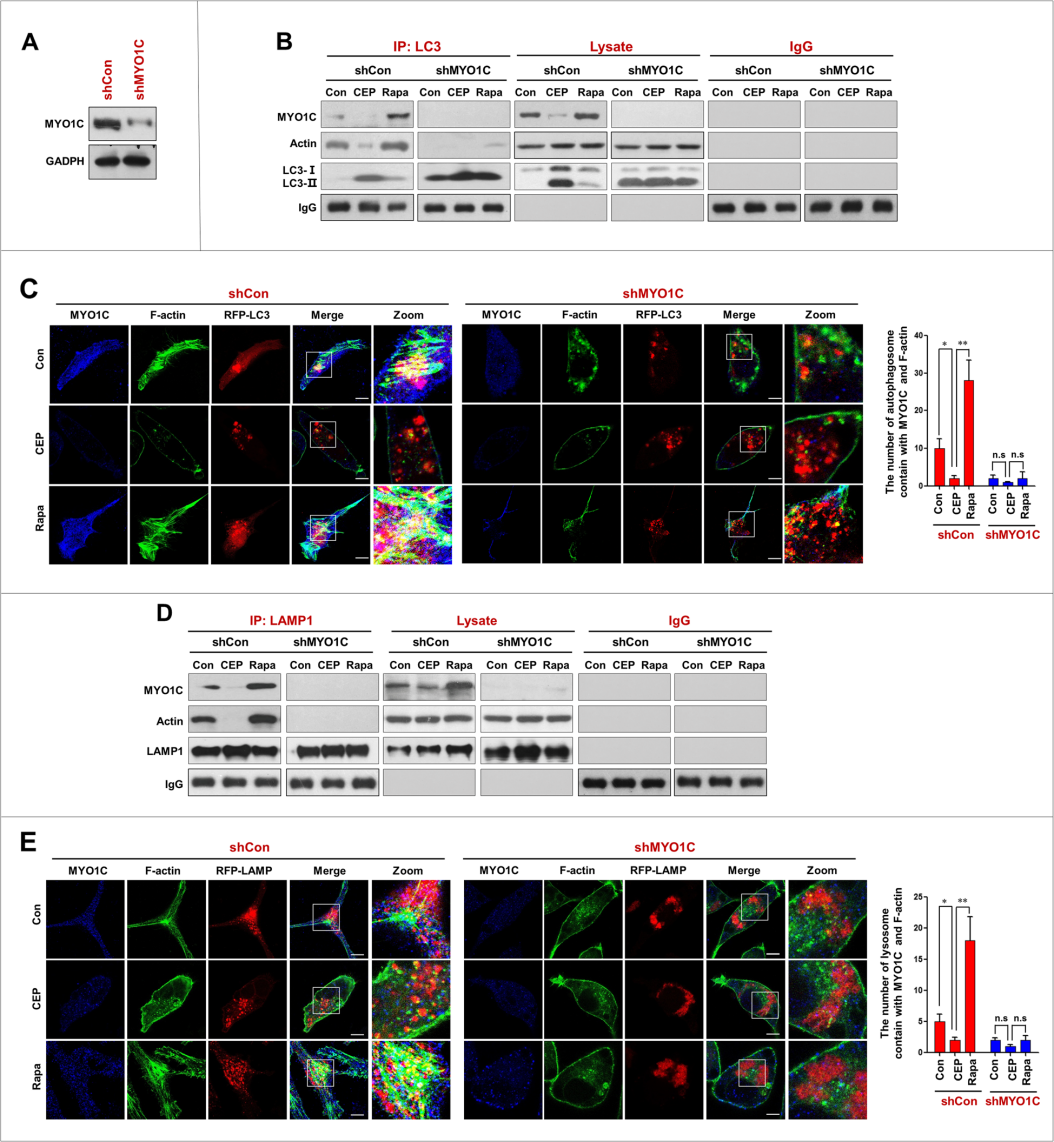
Depletion of MYO1C enhances CEP-inhibited the interaction/ colocalization of MYO1C/F-action with LC3 and LAMP1. **a** MDA-MB-231 cells were transfected with control siRNA (shCon) and MYO1C siRNA (shMYO1C); the expression of MYO1C was determined by western blot. **b** shCon and shMYO1C cells were treated without or with CEP (4 μM) or Rapa (0.25 μM) for 24 h. Whole-cell lysate was prepared and subjected to immunoprecipitation using anti-LC3 and the associated MYO1C and actin were determined using immunoblotting. **c** The fluorescent images of both shCon and shMYO1C cells immunostained for MYO1C (blue), F-actin (green) and LC3 (red) The number of LC3 puncta colocalized with MYO1C and F-actin was quantified from 50 cells in three dependent experiments, data was present as mean ± SD (n.s, not significant; *P < 0.05; **P < 0.01). **d** LAMP1 was immunoprecipitated, and the expressions of MYO1C and actin were determined by immunoblotting. **e** The fluorescent images of both shCon and shMYO1C cells immunostained for MYO1C (blue), F-actin (green) and LAMP1 (red). The number of LAMP1 puncta colocalized with MYO1C and F-actin was quantified from 50 cells in three dependent experiments, data was present as mean ± SD (n.s, not significant; *P < 0.05; **P < 0.01)

To further confirm that downregulation of MYO1C is involved in the CEP-mediated blockade of autophagosome-lysosome fusion, a genetic approach utilizing MYO1C shRNA was employed (Fig. 6a). Immunoprecipitation analysis showed that depletion of MYO1C with shRNA significantly decreased the interaction of LC3 with MYO1C and actin in cells treated with or without CEP and rapamycin (Fig. 6b). Similarly, MYO1C depletion further decreased the colocalization of LC3 with MYO1C and F-actin in cells treated with or without CEP and rapamycin (Fig. 6c). We also investigated the effects of MYO1C depletion on the interaction and colocalization of LAMP1 with MYO1C and F-actin. MYO1C depletion further decreased the interaction and colocalization of LAMP1 with MYO1C and F-actin in cells treated with or without CEP and rapamycin (Fig. 6d and e).

**Fig. 7:**
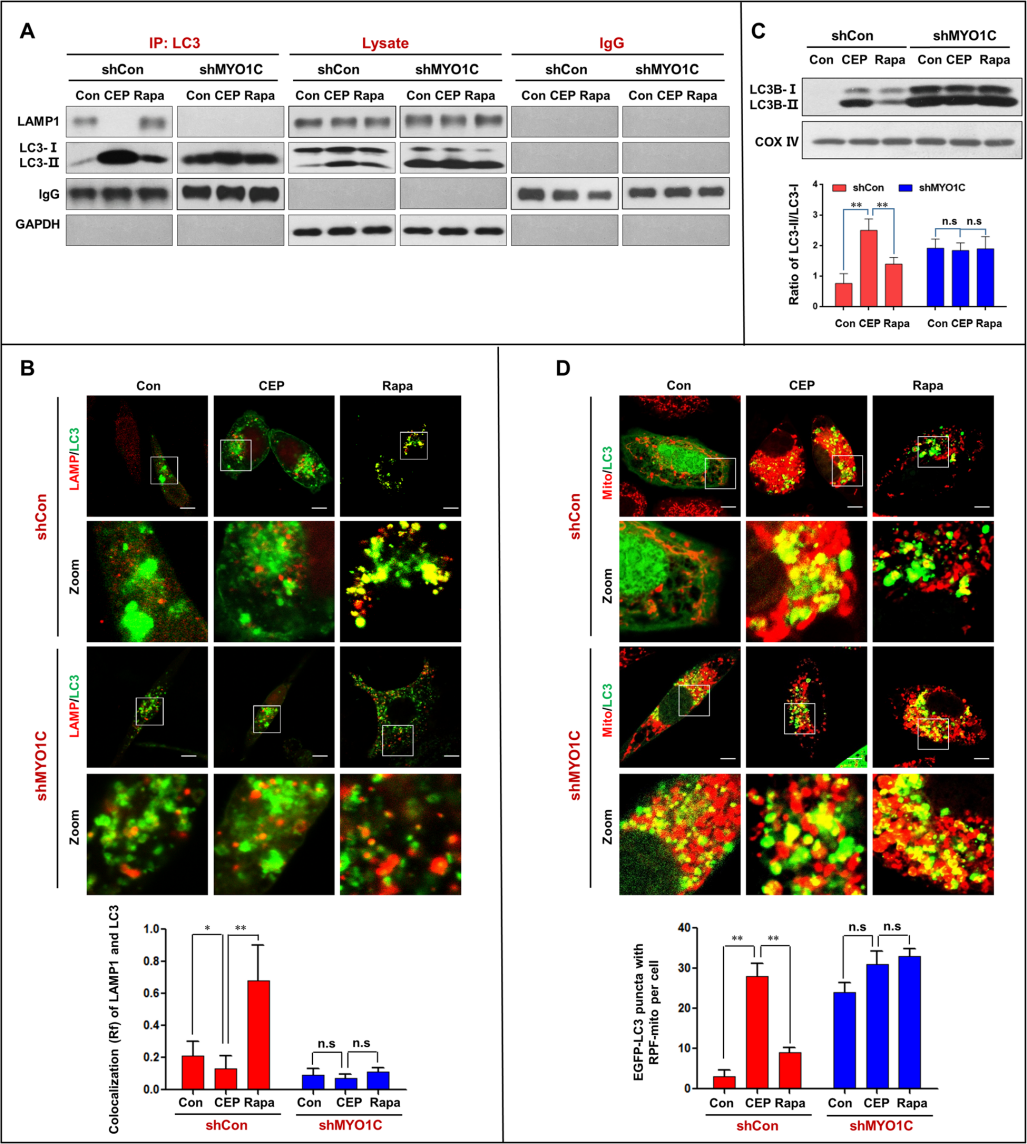
Depletion of MYO1C enhances CEP-mediated blockade of autophagosome-lysosome fusion and the accumulation of mitophagosomes. shCon and shMYO1C cells were treated without or with CEP (4 μM) or Rapa (0.25 μM) for 24 h. **a** Whole-cell lysate was prepared and subjected to immunoprecipitation using anti-LC3 and the associated LAMP1 was determined using immunoblotting. **b** The fluorescent images showed the colocalization of LC3 (red) and LAMP1 (green). The Pearson’s correlation coefficient (R^2^) of LC3 and LAMP1 colocalization was from 50 cells of three independent experiments (mean ± SD, n.s, not significant; *P < 0.05; **P < 0.01). **c** The mitochondrial fraction was prepared and subjected to western blot using antibody against LC3. The ratio of LC3-II/LC3-I was quantified in three independent experiments (mean ± SD, n.s, not significant; **P < 0.01). **d** The fluorescent images of both shCon and shMYO1C cells transfected with EGFP-LC3 (green) and RFP-Mito (red). Quantification of EGFP-LC3 and RFP-mito colocalization puncta in 50 cells of three independent experiments (mean ± SD, n.s, not significant; **P < 0.01)

We next examined the effects of MYO1C depletion on autophagosome-lysosome fusion and accumulation of mitophagosomes in cells treated with either CEP or rapamycin. MYO1C depletion markedly enhanced the inhibition of the interaction and colocalization of LC3 and LAMP1 mediated by CEP treatment and abrogated the effects mediated by rapamycin treatment (Fig. 7a and b). Furthermore, MYO1C depletion further promoted the accumulation of LC3B-II in mitochondria of cells treated with or without CEP and rapamycin (Fig. 7c). Finally, MYO1C depletion markedly increased the accumulation of mitophagosomes in cells treated with or without CEP and rapamycin (Fig. 7d). Taken together, these findings indicate that MYO1C depletion enhances the CEP-mediated blockade of autophagosome-lysosome fusion and the accumulation of mitophagosomes by inhibiting the interaction/colocalization of the MYO1C/F-actin network with either autophagosomes or lysosomes.

### Overexpression of MYO1C promotes autophagosome-lysosome fusion and decreases accumulation of mitophagosomes

**Fig. 8:**
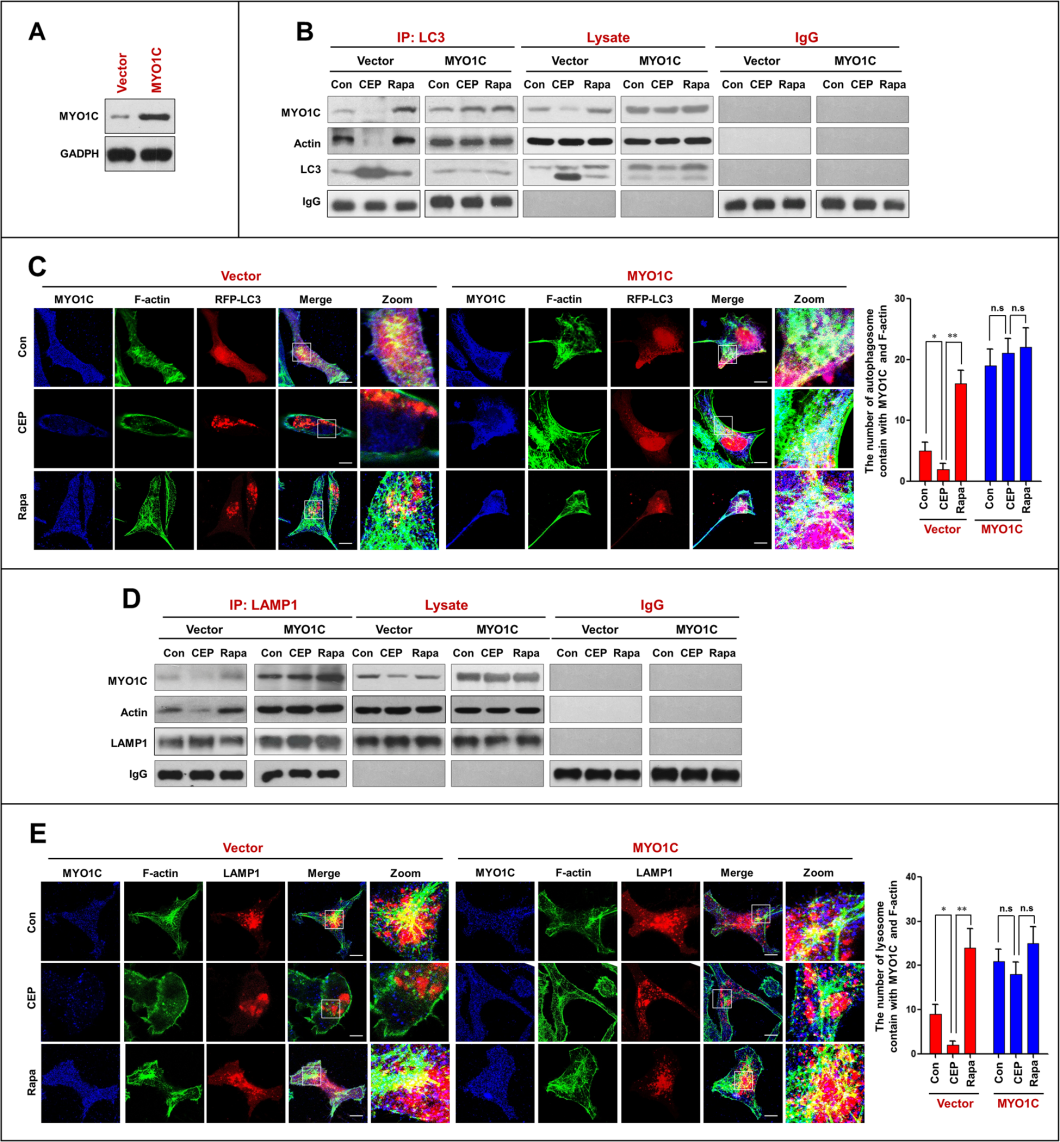
Overexpression of MYO1C promotes the interaction/colocalization of MYO1C/F-actin with LC3 and LAMP1. **a** MDA-MB-231 cells were transfected with control plasmid (Vector) or MYO1C overexpressing plasmid (MYO1C) for 48 h, and the expressing of MYO1C was determined by western blot analysis. Vector or MYO1C cells were treated with CEP (4 μM) and Rapa (0.25 μM) for 24 h. **b** Whole-cell lysate was prepared and subjected to immunoprecipitation using anti-LC3 and the associated MYO1C and actin were determined using immunoblotting. **c** The fluorescent images of cells immunostained for MYO1C (blue), F-actin (green) and LC3 (red). **d** LAMP1 was immunoprecipitated, and the expressions of MYO1C and actin were determined by immunoblotting. The number of LC3 puncta colocalized with MYO1C and F-actin was quantified from 50 cells in three dependent experiments, data was present as mean ± SD (n.s, not significant; *P < 0.05; **P < 0.01) **e** The fluorescent images of both vector and MYO1C cells immunostained for MYO1C (blue), F-actin (green) and LAMP1 (red). The number of LAMP1 puncta colocalized with MYO1C and F-actin was quantified from 50 cells in three dependent experiments, data was present as mean ± SD (n.s, not significant; *P < 0.05; **P < 0.01)

To further assess the functional significance of MYO1C downregulation in the CEP-mediated inhibition of autophagosome-lysosome fusion, a plasmid construct encoding MYO1C was employed. Transfection of MDA-MB-231 cells with the MYO1C overexpression plasmid resulted in a marked increase in the levels of MYO1C (Fig. 8a). Immunoprecipitation analysis showed that overexpression of MYO1C increased the interaction of LC3 with MYO1C and actin in cells treated with or without CEP and rapamycin (Fig. 8b). Similarly, overexpression of MYO1C increased the colocalization of LC3 with MYO1C and F-actin in cells treated with or without CEP and rapamycin (Fig. 8c).

We also investigated the effects of MYO1C overexpression on the interaction and colocalization of LAMP1 with MYO1C and F-actin. As shown in Fig. 8d and e, overexpression of MYO1C increased the interaction and colocalization of LAMP1 with MYO1C and actin in cells treated with or without CEP and rapamycin.

**Fig. 9:**
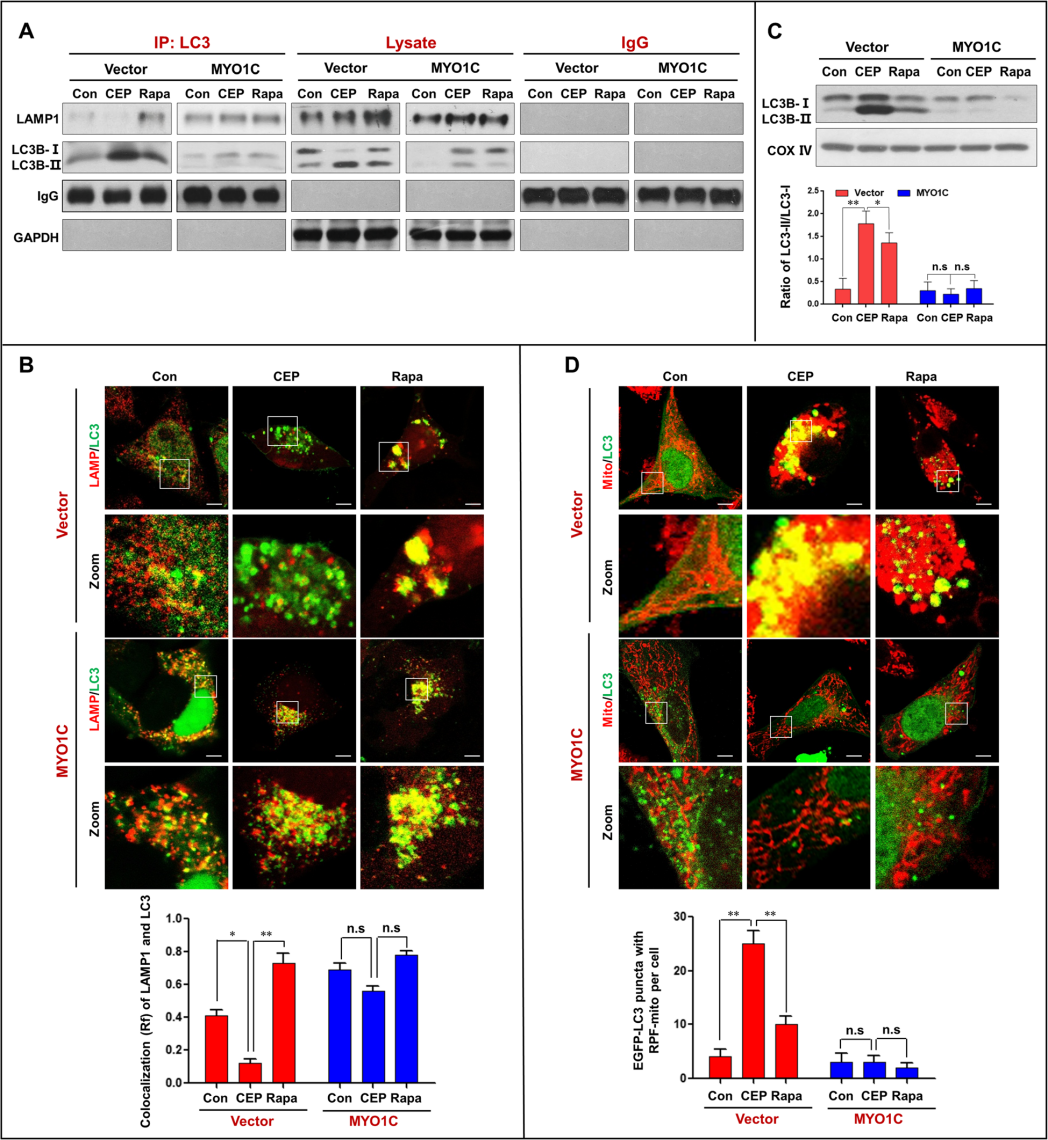
Overexpression of MYO1C promotes the interaction/colocalization of LC3 and LAMP1, and reduced the accumulation of mitophagosomes. The vector and MYO1C cells were treated without or with CEP (4 μM) or Rapa (0.25 μM) for 24 h. **a** Whole-cell lysate was prepared and subjected to immunoprecipitation using anti-LC3 and the associated LAMP1 was determined using immunoblotting. **b** The fluorescent images showed the colocalization of LC3 (red) and LAMP1 (green). The Pearson’s correlation coefficient (R^2^) of LC3 and LAMP1 colocalization was from 50 cells of three independent experiments (mean ± SD, n.s, not significant; *P < 0.05; **P < 0.01). **c** The mitochondrial fraction was prepared and subjected to western blot using antibody against LC3. The ratio of LC3-II/LC3-I was quantified in three independent experiments (mean ± SD, n.s, not significant; *P < 0.05 or **P < 0.01). **d** The fluorescent images of both vector and MYO1C cells transfected with EGFP-LC3 (green) and RFP-Mito (red). Quantification of EGFP-LC3 and RFP-mito colocalization puncta in 50 cells of three independent experiments (n.s, not significant; **P < 0.01)

Next, we examined the effects of MYO1C overexpression on autophagosome-lysosome fusion and accumulation of mitophagosomes in cells treated with either CEP or rapamycin and found that overexpression of MYO1C increased the interaction and colocalization of LC3 and LAMP1 in cells treated with or without CEP and rapamycin (Fig. 9a and b). Last, we examined the effects of MYO1C overexpression on the accumulation of mitophagosomes and found that overexpression of MYO1C significantly decreased the accumulation of mitophagosomes in cells treated with or without CEP and rapamycin (Fig. 9c and d). Taken together, these findings further highlight a critical role of MYO1C in autophagosome-lysosome fusion. Overexpression of MYO1C promoted the autophagosome-lysosome fusion inhibited by CEP by restoring the interaction/colocalization of the MYO1C/F-actin network with either autophagosomes or lysosomes.

## Discussion

In the present study, we provide definitive evidence that CEP triggers the accumulation of autophagosomes/mitophagosomes by blockade of autophagosome-lysosome fusion. The accumulation of autophagosomes could be caused by either (1) reduced proteolytic activity of the lysosomal enzymes by changing lysosomal pH, (2) a defect in the maturation of lysosomal cathepsins, or (3) impaired autophagosome fusion with the lysosome. We investigated these different possibilities and found no obvious defect in the proteolytic activity of the lysosomal enzymes (i.e., LAMP1 and LAMP2) or any changes in lysosomal pH, suggesting that lysosomal activity is not necessary for the accumulation of autophagosomes/mitophagosomes mediated by CEP. In this study, we found that CEP treatment could markedly decrease the interaction and colocalization of LAMP1 (lysosome) and LC3 (autophagosome). This leads us to raise the possibility that a blockade of autophagosome-lysosome fusion contributes to the accumulation of autophagosomes/mitophagosomes mediated by CEP.

Increasing evidence has revealed that several actin-based motor proteins, including myosin I, II and VI, have been implicated in autophagy [17]. Class I myosin functions as a linker between actin cytoskeletal proteins and membranes in several cellular processes. MYO1C has a head domain (single motor domain), which binds actin or nucleotides, and a membrane-binding tail domain [35]. Previous studies have shown that MYO1C influences numerous cellular processes. For example, MYO1C leads G-actin transport during cell migration and intracellular membrane trafficking [18], and MYO1C can link the actin cytoskeleton to fused cortical granules and transduce the force generated by the actin cytoskeleton, which can compress the vesicle membrane [36]. It has recently been reported that loss of functional MYO1C disrupts autophagosome-lysosome fusion by disturbing the cellular distribution of lipid rafts, leading to the accumulation of autophagosomes [15]. Consistent with this report, our study indicated that the downregulation of MYO1C mediated by CEP could contribute to blockade of autophagosome-lysosome fusion and accumulation of mitophagosomes based on the following findings. First, CEP treatment caused dose- and time-dependent downregulation of MYO1C. Second, the interaction/colocalization of MYO1C with either LC3 or LAMP1 was reduced by CEP treatment. Third, knockdown of MYO1C markedly enhanced the inhibition of the interaction/colocalization of LC3 and LAMP1 mediated by CEP treatment and abrogated these effect mediated by rapamycin treatment. Fourth, knockdown of MYO1C further promoted the accumulation of mitophagosomes mediated by CEP or rapamycin. Finally, overexpression of MYO1C promoted autophagosome-lysosome fusion and inhibited the accumulation of mitophagosomes mediated by either CEP or rapamycin. Such findings highlight the importance of MYO1C in mediating autophagosome-lysosome fusion.

Actin is an evolutionarily conserved molecule that self-assembles into long polymers. Actin filament assembly and disassembly provide mechanical forces for a wide range of cellular activities that involve membrane deformation, such as cell motility, phagocytosis, endocytosis and cytokinesis [37]. Recent evidence has revealed that the actin cytoskeleton is required for membrane fusion and may play an essential role in autophagy [32, 38, 39]. Because MYO1C is an actin filament transporter that mediates actin filament movement to the cellular membrane [11, 34], we hypothesize that MYO1C may promote autophagosome–lysosome fusion through remodeling of the actin cytoskeleton. Supporting this notion, our results highlight the importance of MYO1C in mediating autophagosome-lysosome fusion through remodeling of the actin cytoskeleton. First, the interaction/colocalization of LC3 with either MYO1C or F-actin was decreased by CEP treatment. Similarly, the interaction/colocalization of LAMP1 with either MYO1C or F-actin was also decreased by CEP treatment. Second, knockdown of MYO1C significantly abrogated the interaction/colocalization of either LC3 or LAMP1 with MYO1C and F-actin. Third, overexpression of MYO1C promoted the interaction/colocalization of either LC3 or LAMP1 with MYO1C and F-actin. Thus, these findings suggest that the function of MYO1C in the remodeling of the actin cytoskeleton may have an important impact on autophagosome-lysosome fusion.

## Conclusion

**Fig. 10:**
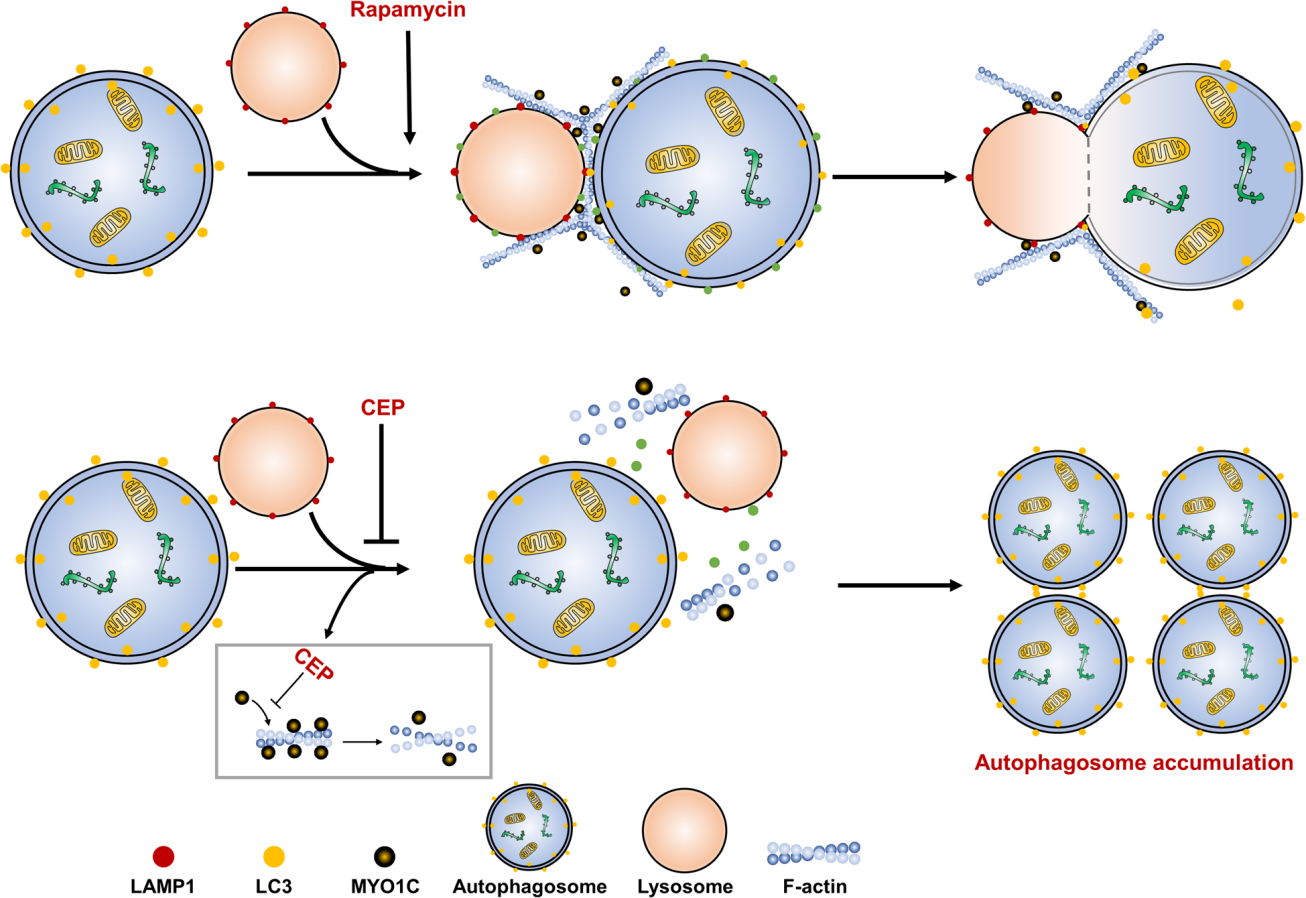
A hypothetical model of CEP-triggered the accumulation of mitophagosomes through blockade of autophagosome-lysosome fusion. CEP triggers downregulation of MYO1C, leading to disrupting the recruitment of MYO1C and F-actin to autophagosomal and lysosomal membranes, resulting in blockade of autophagosome-lysosome fusion, and culminating in the accumulation of mitophagosomes

This study clearly demonstrates that CEP triggers the accumulation of mitophagosomes through blockade of autophagosome-lysosome fusion. Our findings highlight a key role of MYO1C in the regulation of autophagosome-lysosome fusion. These findings support a hypothetical model of CEP-triggered accumulation of mitophagosomes through blockage of autophagosome-lysosome fusion (Fig. 10). In this model, CEP effectively induces downregulation of MYO1C, leading to disruption of the recruitment of MYO1C and F-actin to autophagosomes and lysosomal membranes, resulting in blockade of autophagosome-lysosome fusion and culminating in the accumulation of mitophagosomes. Thus, this novel autophagosome-lysosome fusion machinery could be an attractive therapeutic target for developing novel therapies for cancer treatment. Our findings also suggest that CEP could potentially be further developed as a novel autophagy/mitophagy inhibitor, and a combination of CEP with classic chemotherapeutic drugs could represent a novel therapeutic strategy for the treatment of breast cancer.

## Supplementary information

**Additional file 1: Table S1**. (XLSX 130 kb)

## Data Availability

All data generated or analyzed during this study are included in this published article and its supplementary information files.

## Abbreviations

ATG: autophagy-related
Baf: bafilomycin A_1_
CEP: cepharanthine
EGFP: enhanced green fluorescent protein
HDAC6: histone deacetylase-6
INPP5E: inositol polyphosphate-5-phosphatase E
LAMP1: lysosomal-associated membrane protein1
LC3: microtubule-associated protein 1 light chain 3
mRFP: modified red fluorescent protein
MYO1C: myosin 1 C
Rapa: rapamycin
siRNA: small interfering RNA
SNARE: soluble N-ethylmaleimide-sensitive factor attachment protein receptor
SQSTM1: sequestosome 1
